# Analysis and improvement of sports industry development and public health strategy under low-carbon economic structure

**DOI:** 10.3389/fpubh.2023.1152452

**Published:** 2023-03-21

**Authors:** Wenhao Zhang, Chuan Mou

**Affiliations:** ^1^Institute of Physical Education, Kunsan National University, Gunsan, Jeollabuk-do, Republic of Korea; ^2^Jilin Institute of Physical Education, College of Physical Education, Changchun, Jilin, China; ^3^Institute of Physical Education, Sichuan University, Chengdu, Sichuan, China

**Keywords:** low carbon economy, sports industry, public health, GDP, sustainable development

## Abstract

With the continuous development of society, various industries are rising and developing rapidly. Against this background, the energy crisis has come quietly. Therefore, to improve the quality of life of residents and promote the comprehensive and sustainable development of society, it is essential to enhance the development of the sports industry and formulate public health strategies under the background of a low carbon economy (LCE). Based on this, to promote the low-carbon development of the sports industry and optimize the formulation of social public health strategies, firstly, this paper introduces the low-carbon economic structure and its role in society. Then, it discusses the development of the sports industry and the necessity of perfecting public health strategy. Finally, based on LCE’s development background, the sports industry’s development situation in the whole society and M enterprises is analyzed, and suggestions are put forward to improve the public health strategy. The research results show that the current development prospect of the sports industry is extensive, and the added value of the sports industry will be 1,124.81 billion yuan in 2020, up by 11.6% year-on-year, accounting for 1.14% of Gross Domestic Product (GDP). Although industrial development declined in 2021, the added value of the sports industry accounts for an increasing proportion of GDP yearly, which shows that the sports industry is playing an increasingly important role in economic growth. And through the analysis of the development of M enterprise sports industry as a whole and in different directions, this paper shows that enterprises should reasonably control the development of various industries to provide impetus for the overall development of enterprises. The innovation of this paper lies in the innovative use of the sports industry as the primary research object, and its development under LCE is studied. This paper not only supports the sustainable development of sports industry in the future, but also contributes to improving public health strategy.

## 1. Introduction

Under the background of social evolution, the demand for energy in all sectors of society is also increasing, and the energy crisis caused by it is also deepening. Therefore, in order to promote the sustainable development of society and improve the utilization rate of energy, the concept of low-carbon development came into being. Under the theoretical framework of sustainable development economics, low carbon economy (LCE) should be the economy with the lowest carbon emission, ecological environment cost and socio-economic cost, and it is an economy with strong ecological sustainability that can improve the self-regulation ability of the earth’s ecosystem (1). The image generalization and realistic form of low-carbon economic development is a theory of low-carbon economic development (2). Developing LCE is an urgent requirement and strategic task to promote scientific development, and the key is to carry out the ecological revolution of the energy economy (3). Developing LCE and realizing low-carbon development is a systematic project to develop LCE. Based on this, the development of many social industries tends to the concept of low-carbon development. As one of the pillar industries of society, the sports industry not only plays an essential role in social development but also provides critical support for improving public health system. Therefore, it is equally important to evaluate the development of the sports industry under the background of low-carbon economic development, which is necessary for improving public health strategies, and many researchers have studied it at present.

Under the current low-carbon economic structure, how to develop various industries has also become an essential issue in the recent social reform. Among them, the sports industry, as one of the pillar industries in society, has a far-reaching impact on the public. Therefore, developing the sports industry under the low-carbon economic structure is also necessary. There are many defects in the current sports industry, the most serious of which is that the structure of the sports industry is seriously flawed, and its development is unbalanced and unscientific. It only pays attention to a certain sporting goods industry, sports clothing industry and other specific industries and does not pay enough attention to and invest in some future core industries and new industries, including the sports animation industry and sports information industry, which has seriously affected the all-round development of the sports industry. Based on this, to promote the comprehensive development of the sports industry, how to develop the sports industry reasonably under the low-carbon economic structure and thus promote public health is a significant social problem.

Based on this, this paper first discusses the theory of sports industry development and public health under the low-carbon economic structure. Then the relationship between the development of the sports industry and public health is expounded. Finally, based on the background of green economy development, the future effect of the sports industry is studied. The innovation of this paper is to check the development of the sports industry under the new situation of LCE and analyze its future development strategy. This paper not only supports the sustainable development of the sports industry, but also contributes to improving the social public health system.

## 2. Literature review

The emergence of the concept of low Carbon economy has significantly impacted social development, which can be found in many research works. Semieniuk et al., Wang et al. (4) contended that climate change had become the most popular topic worldwide for profound and complex reasons. From the perspective of the earth’s formation and the interaction between living matter and dead matter, the air is the first element to create and maintain life. Their research finding implied climate change was a top priority for the human environment. Accordingly, in today’s increasingly severe environmental pollution, vigorously developing LCE has become a crucial task of society (4). Keshkar et al. (5) stated that the sports industry was the sum of similar economic sectors offering sports products for social demands. The sports industry was integral to the national economy and industrial system. Developing the sports industry in line with the law of the sports economy was conducive to international standards with the classification method. As a new growth point of the national economy, the sports industry has already embraced preliminary conditions.

Nevertheless, there were also many difficulties (5). Weight et al. (6) warranted the sports industry’s substantial role in improving public health strategy (PHS). At the same time, to catch up with LCE, continuously optimizing the sports industry was extremely important. Hence, to maintain a sustainable sports industry and promote PHS, optimizing the sports industry by extending the LCE structure could help increase social development points (6).

As far as the above literature review is concerned, the development of LCE is relatively mature. Under the background of LCE, it is urgent to optimize the development strategy of the sports industry to better conform to the concept of low-carbon development. At present, the research on low-carbon economic structure only stays in the study on its development and the effect of its application in some narrow structural levels. Still, there is little research on its overall social development level. Therefore, to reasonably reveal the influence of low-carbon economic structure on the whole society, this paper designs to study the impact of the development of the sports industry on the healthy development of the social public under the low-carbon economic structure to explore the influence effect of the development of low-carbon economic system on the whole society.

## 3. Development of sports industry under LCE

### 3.1. LCE structure

As an economic term, LCE was formally proposed by the British government in 2003 in the context of climate change concerns and the need to maintain national energy security (7). Since then, LCE has received close attention from the international community. The development mode of LCE is a historical progress trend and a new social and economic form of the postindustrial society in the transition from traditional industrial civilization to a higher ecological civilization (8). Currently, research on trade problems caused by climate change mainly involves trade carbon leakage and trade-implied carbon, border tax adjustment & carbon tariff, carbon footprint accounting standards, and carbon label certification system, carbon trading, and clean development mechanism (9). They are explained in detail below.

#### 3.1.1. Carbon leakage

It is defined under the United Nations Framework Convention on Climate Change. Carbon leakage occurs when high-carbon emission industries and enterprises in industrialized countries (usually with strict low-carbon environmental regulations) transfer the high-carbon emissions production to developing countries. The developing nation usually lacks mandatory emission reduction obligations. As a result, governments, and regions with loose carbon emission regulations see a substantial rise in carbon emissions, resulting in “false” carbon emission reduction in developed countries. That is to say, the problem of carbon emissions in developing countries has become more serious, even causing carbon emissions to increase globally. Usually, the carbon leakage degree is usually calculated and measured by the ratio of the carbon dioxide (CO_2_) emissions ratio of countries with higher domestic emission reduction and governance actions outside their borders to their domestic CO_2_ emission reductions (10).

#### 3.1.2. Trade-implied carbon

Trade-implied carbon is a fundamental concept in studying the relationship between international trade and LCE from the perspective of trade and the environment. Although trade-implied carbon is a new concept in the research of LCE, the implied concept has a long history. For a long time, the implicit energy and implicit carbon emissions of trade products have been ignored in traditional international trade research. Until recently, with the growing concern of the international community about global warming, the implicit energy and implicit carbon emissions of international trade have been gradually taken seriously (11).

#### 3.1.3. Trade and carbon accounting

Carbon accounting refers to accounting for Greenhouse Gas (GHG) emissions in the production process of enterprises. Carbon accounting mainly targets major carbon emission bodies like enterprises. Thus, scientific carbon accounting can help identify the weak links between energy utilization and environmental protection in enterprises or product manufacturing. Thereby, it encourages enterprises to adopt Energy-Conservation and Emission Reduction (ECER) technologies and improve management measures. Ultimately, their international competitiveness can be strengthened (12).

#### 3.1.4. Trade and carbon footprint labeling

A carbon footprint label is the main form of low-carbon product certification. It refers to the accounted GHG emissions in the production process of products. It marks the carbon footprint on the product label exponentially to inform consumers of the carbon information of products and guide them to choose low-carbon products to achieve ECER (13).

#### 3.1.5. Trade and border tax adjustments

The border tax adjustment and other measures have been proposed to comply with the climate change policy. For the non-cooperative cross-border pollution problem, it will be the best policy choice to levy import tariffs on illegally polluting countries (14).

#### 3.1.6. Trade and carbon tariffs

Carbon tariff refers to the special tariff on CO_2_ emissions of imported energy-intensive products with high pollution proposed by Europe, America, and other developed countries (15).

With the development of LCE, the identification of carbon emission responsibility in international trade has increasingly become the focus of debate in international climate negotiations (16). There are two main criteria for determining the responsibility of international trade for carbon emissions: the producer responsibility principle that directly causes pollution, and the consumer responsibility principle that causes pollution (17). Obviously, developed countries can falsely reduce the total carbon emissions reported in China by replacing domestic production with imports, but the generation of carbon leakage does not help to reduce global greenhouse gas emissions (18). Therefore, in the future international climate negotiations, the accounting of greenhouse gasses in a country should not be limited to the national boundaries, but should be comprehensively considered from the production and consumption of goods or services to redefine the carbon emission responsibility. At present, the research on trade carbon emissions is changing from producer responsibility to consumer responsibility, which may lead to a new global carbon reduction strategy, so it must be paid full attention (19). The low-carbon economic structure formed on this basis has also become an important reference index for the development of various industries in the future.

Low-carbon economic structure is the basic industrial structure based on the development of LCE. From the relationship point of view, different industries influence and promote each other, which has something in common and provides a basic guarantee for industrial structure adjustment. At present, the general trend of the overall industrial structure of LCE is developing from the traditional structure of agriculture and industry to the tertiary industry such as service industry and tourism. Meanwhile, in the internal development process of various industries, the working methods and operation modes are constantly optimized. For example, when the agricultural industry is developing, it has begun to develop from the traditional extensive business model to the intensive and refined direction, which makes the limited land resources play a greater role and create more benefits. Moreover, various agricultural enterprises are also developing in the direction of branding, introducing professional production equipment and integrating regional culture into agricultural products in combination with regional characteristics. This new model improves the social benefits of products on the basis of ensuring the economic benefits of agricultural industry. In addition, in view of the problems of carbon emission and land pollution, agricultural workers have also begun to study ways to save water resources, and conducted in-depth research on the rational selection of fertilizer types and fertilization methods, with the aim of combining land use with land cultivation and promoting the sustainable development of agricultural industry. This scientific management model has effectively alleviated the carbon emission problem to a certain extent and achieved the goal of low-carbon agricultural management. Therefore, how to develop the sports industry in this context is also an important issue.

### 3.2. Development of the sports industry

The sports industry is a new driving force for national economic growth. While providing sports products for society, it also improves the physical quality of residents, enhancing national spirit, achieving social progress, and enhancing international influence (20). As a sunrise industry, sports greatly impact social and economic development and determine citizens’ well-being. It is a rising growth point of China’s social and economic development. Therefore, seizing this new opportunity by following the market rules can cope with the problems and drawbacks in developing the sports industry. For example, alleviating the unbalanced development can China help realize the healthy development of the sports industry and make China an intensely competitive nation (21).

**Figure 1:**
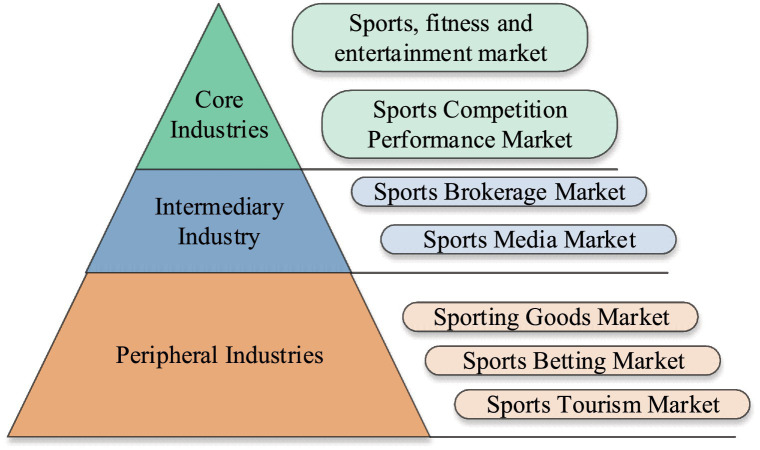
Classification of the sports industry.

The new statistical classification divides China into sports goods and related products manufacturing, sports service, and sports field facilities construction. The sports service industry includes sports management activities, sports competitions & performances, sports fitness & leisure activities, sports venues & facilities management, and other subcategories (22). Figure 1 shows the refined classification of the sports industry.

**Figure 2:**
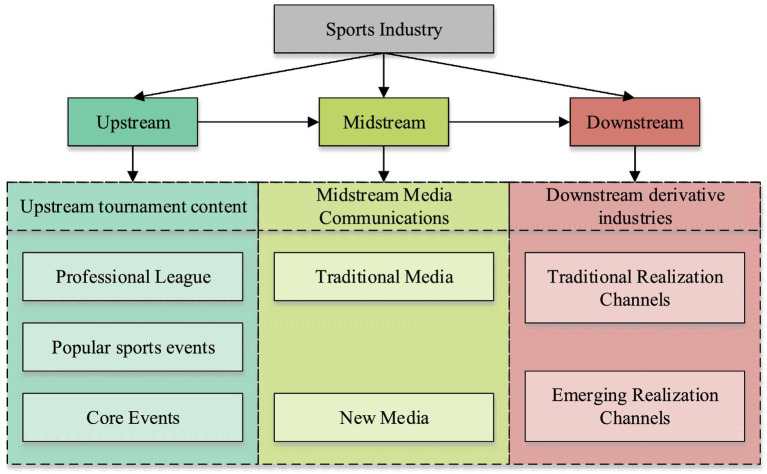
Industrial chain structure of the sports industry.

As per Figure 1, the sports industry has formed three main levels since its development: upstream sports events, midstream sports media, and downstream sports derivative industries (23). Figure 2 shows the industrial chain structure of the sports industry.

In Figure 2, the development of sports industry has been very mature, and it has promoted the development of social industries in a great range. However, under the current background of low-carbon economic development, how to adjust the development direction of sports industry to meet the needs of the times has also become an important task of current social development, so this paper plays an important role in the development of sports industry (24). Sports industry chain refers to a number of enterprises that have upstream and downstream relations and ultimately provide sports products and services to consumers. The essence of sports industry chain is the value chain that provides customer value to sports consumers. The sports industry includes the sports noumenon industry, peripheral industry, sports intermediary industry, and sports industry consumers. Among them, sports noumenon industry is the core of the whole sports industry, including sports competition and mass fitness. The industrial chain of sports peripheral industries includes sports goods suppliers, sports equipment suppliers, sports clothing suppliers, sports tourism suppliers, sports gamblers, sports medical providers and sports builders. The industrial chain of sports intermediary industry includes sports advertisers, sports sponsors and sports insurance industry.

### 3.3. Principles and theories of public health

Public health is an ensemble system. It involves science, art, practical skills, and beliefs, guiding, maintaining, and improving the health of all. It helps the public prevent disease, prolong their life span, and promote a nation’s health and efficiency through organized community efforts (25). Organized community efforts aim to build a healthy environment both physically and mentally. These measures can control diseases, reduce infections, and raise health awareness. Furthermore, organized medical staff provides early diagnosis, prevention, and treatment services. Thereby, social mechanisms can be established to ensure community members’ right to access healthy living standards. Eventually, these beneficiary policies arouse citizens’ birthright: health and longevity (26).

The concept of public health has the following characteristics. (1) Public health is a public social product and a public development project. There must be joint participation and collaboration of governments at all levels, communities, and different departments. (2) Public health considers the whole population as the object and concerns the health of all social members. Public health investigates human health problems collectively or at the group level. It has the goal of eliminating premature deaths and possible diseases. In particular periods such as during epidemic infectious diseases, public health mainly concerns the pathogenesis (27).

Based on personal health, public health looks into the health problems of all social members. By comparison, personal health concerns individual health problems and is the field of personal autonomy (28). From an individual perspective, in addition to genetic factors, personal lifestyles, diet, preferences, and personality often affect their health. Unlike public health, personal health extends from personal responsibility to social responsibility. Moreover, public health protects individuals from the influence of others’ unsanitary or infectious diseases, strives to improve the living environment of social members, and maintains & enhances the health quality of all social members. Of course, public and personal health factors can also act on one another (29).

### 3.4. Designing the research model of sports industry development under the background of LCE

The selection of key industries should follow the benchmark of comparative advantage, correlation effect, diffusion effect, Miyohei Shinohara’s two benchmark theories, technological progress, and employment (30). According to these selection benchmarks, the following corresponding index values are selected for measurement. They are the industrial added-value scale, total capital scale, employment scale, export scale, profit and tax scale, fixed asset output rate, technical level, labor productivity, capital profit and tax rate, and the proportion of scientific research personnel. At the same time, two new low-carbon indicators are introduced to meet the requirements of LCE development: carbon productivity and carbon intensity competitiveness (31). The detailed indicators are explained below (32):

a. Scale of industrial added value:

(1) $$GY_{i}=Y/\sum Y$$

In Equation (1), $GY_{i}$ is the scale of total industrial added value, and Y denotes the total industrial added value.

b. Total capital scale:

(2) $$GZM_{i}=ZM/\sum ZM$$

In Equation (2), $GZM_{i}$ is the total capital scale, and ZM represents the balance of current assets.

c. Employment scale:

(3) $$JL_{i}=L/\sum L$$

Here, $JL_{i}$ stands for the employment scale, and L mean the mean employee number.

d. Export scale:

(4) $$EX_{i}=X/\sum X$$

Here, $EX_{i}$ and X are the export scale and the total export volume.

e. Profit and tax scale:

(5) $$SR_{i}=R/\sum R$$

In Equation (5), $SR_{i}$ and R represent the scale of profits and taxes and the total amount of profits & taxes.

f. Output rate of fixed assets:

(6) $$K_{i}=Y/K$$

Here, $K_{i}$ , Y , and K stand for the output rate of fixed assets, the total added value of production, and the original price of fixed assets, respectively.

g. Technical level:

(7) $$E_{\left(t\right)}=Y_{i\left(t\right)}/K_{i\left(t\right)}^{\partial }L_{i\left(t\right)}^{\rho }$$

In Equation (7), $E_{\left(t\right)}$ , $Y_{i\left(t\right)}$ , and t indicates the technical level, the total output value, and the year, respectively. $L_{i\left(t\right)}^{\rho }$ and $K_{i\left(t\right)}^{\partial }$ represent the mean employee number and the total amount of funds, respectively.

h. Labor productivity:

(8) $$Q_{i}=\Delta Y_{i}/L_{i}$$

In Equation (8), $Q_{i}$ , $\Delta Y_{i}$ , and $L_{i}$ denote the labor productivity, the increase in output value, and the number of workers, respectively.

i. Capital profit & tax rate:

(9) $$\delta_{i}=R_{i}/K_{i}$$

Here, $\delta_{i}$ , $R_{i}$ , and $K_{i}$ are the capital profit & tax rate, the total profit & tax, and the total amount of funds, respectively.

j. Proportion of scientific research personnel:

(10) $$A_{i}=M_{i}/L_{i}$$

Here, $A_{i}$ , $M_{i}$ , and $L_{i}$ represent the proportion of scientific researchers, the total number of scientific researchers, and the number of workers, respectively.

k. Carbon productivity:

(11) $$C_{i}^{\mathrm{pro}}={\left(\Delta Y/E_{{co}_{2}}\right)}_{i}$$

In Equation (11), $C_{i}^{\mathrm{pro}}$ , ΔY , and $E_{{co}_{2}}$ mean the carbon productivity, industrial added value, and the CO_2_ emission, respectively. Equation (12) calculates $E_{{co}_{2}}$ :


(12) $$E_{{CO}_{2}}=\sum {\left(M\times C_{{CO}_{2}}\right)}_{n}$$

Here, M and $C_{{CO}_{2}}$ are the energy consumption and the carbon emission coefficient corresponding to energy.

l. Carbon strength competitiveness:

(13) $$C_{i}^{int}={\left(C_{i}^{\mathrm{pro}}\right)}_{\mathrm{region}}/{\left(C_{i}^{\mathrm{pro}}\right)}_{\mathrm{nation}}$$

In Equation (12), $C_{i}^{int}$ , ${\left(C_{i}^{\mathrm{pro}}\right)}_{\mathrm{region}}$ , and ${\left(C_{i}^{\mathrm{pro}}\right)}_{\mathrm{nation}}$ represent the carbon strength competitiveness, the regional carbon productivity, and the national carbon productivity, respectively. These indicators can measure the extent to which an industry’s carbon emission technology is ahead of the national average.

## 4. Experimental design and performance evaluation

### 4.1. Datasets collection

**Figure 3:**
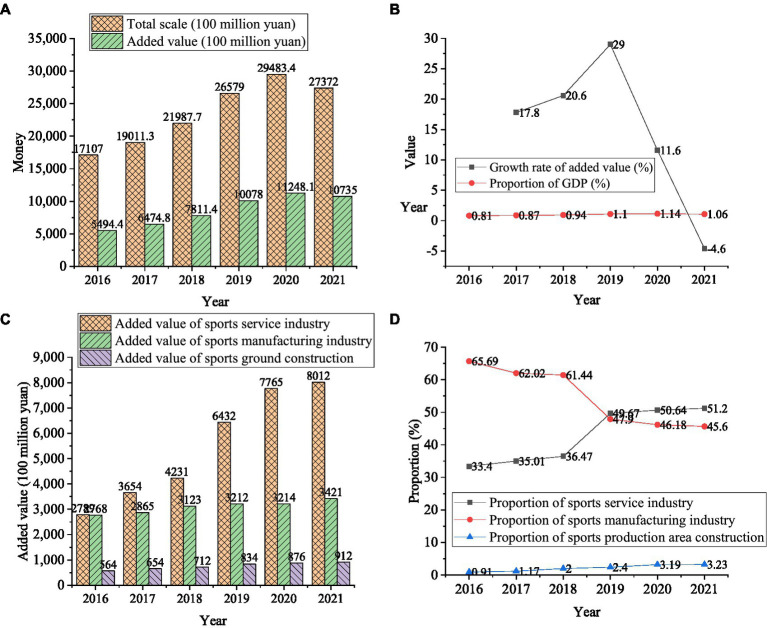
Statistics on the development of China’s sports industry **(A)** refers to the overall development of the sports industry, **(B)** is the overall growth rate of the sports industry, **(C)** illustrates the development of the sports industry in different directions, and **(D)** shows the proportion of development in different directions.

Part of the data sources studied in this paper are the annual sports industry announcement data published by China National Sports Bureau, including the sports industry development data from 2016 to 2021. This paper collects and analyzes these data, and obtains the results shown in Figure 3. In addition, this paper also takes M sports enterprises as the main research object. M enterprise is a leading scientific sports service provider in China. It was founded in 1996 and listed on the main board of Shanghai Stock Exchange in 2020. The company’s main business is the research and development, production and sales of fitness equipment and display shelf products, including indoor fitness equipment and outdoor path products. At present, the company has formed a complete business system of fitness equipment and display shelf products. The company has achieved a leading position in the fitness equipment industry with years of manufacturing experience, product supply system, market-oriented product design, excellent product quality, and perfect supporting service capabilities. The above data sources are statistically analyzed by SPSS, and then processed and analyzed by the above formula to explore the development of sports industry.

M Enterprise is committed to providing users with professional and scientific fitness solutions, products, and services, integrating simple sports into everyone’s life. It helps people build a healthy lifestyle to reach their healthcare goals, promoting a better and harmonious society.

M Enterprise was established in China’s sports industry base. It has thousands of service outlets nationwide and has established an integrated online and offline service sales channel. The service sales system is almost perfect. At the same time, M enterprise prioritizes the international brand strategy. Its business involves more than 70 countries and regions, such as Europe, America, the Middle East, and Southeast Asia.

### 4.2. Performance evaluation

#### 4.2.1. Analysis of the development of China’s sports industry

This section statistically analyzes the overall development of China’s sports industry through data collection to provide a basic reference for studying the sports industry development of M enterprises. It is expected to provide support for exploring the overall development of the industry and a reference for improving future PHS. Figure 3 shows the statistical results of the overall development of China’s sports industry.

As shown in Figure 3, the added value of the sports industry was 549.44 billion RMB in 2016 and 647.48 billion Ren Min Bi (RMB) in 2017, a year-on-year (YoY) increase of 17.8%, accounting for 0.87% of the gross domestic product (GDP). In 2018, the added value of the sports industry was 781.14 billion RMB, with a YoY growth of 20.6%, accounting for 0.94% of GDP. In 2019, the added value of the sports industry was 1,007.8 billion RMB, with a YoY growth of 29.0%, accounting for 1.1% of GDP. In 2020, the added value of the sports industry was 1,124.81 billion RMB, up 11.6% YoY, accounting for 1.14% of GDP. Although industrial development declined in 2021, the proportion of the added value of the sports industry in GDP increased YoY. The result indicates that the sports industry is increasingly important in China’s economic growth.

#### 4.2.2. Analysis of the development of the sports industry in M enterprises under LCE

**Figure 4:**
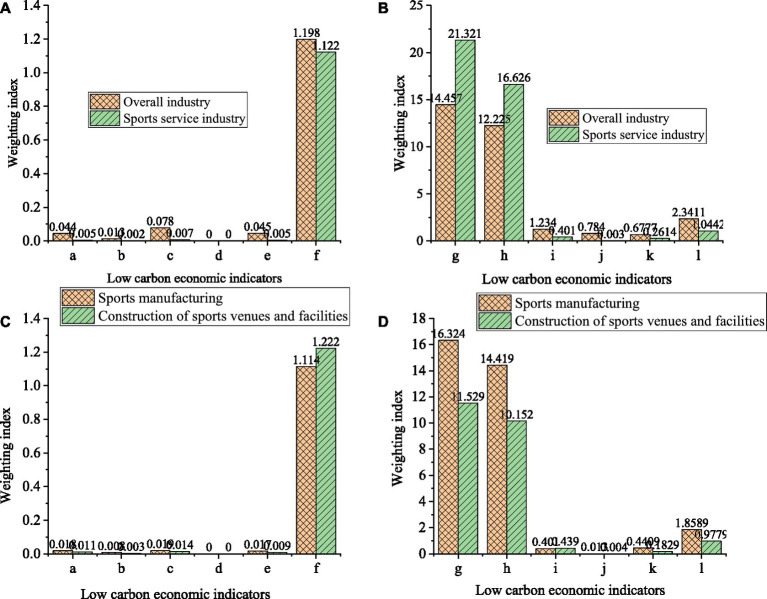
Development status of the sports industry and M enterprise **(A,B)** refer to the overall sports industry and sports service industry, and **(C,D)** is the sports manufacturing industry and the construction of sports venues and facilities.

Low carbon economy is an important indicator of current social development. This section evaluates the development of the sports industry and M Enterprise through various indicators of LCE. It is hoped to provide a reference for the future development of enterprise and support for social PHS. Figure 4 shows the evaluation results of Enterprise M’s sports industry development based on LCE.

In Figure 4, a-l in the figure refers to the above-mentioned low-carbon economic indicators. Through the analysis of the development of the sports industry as a whole and in different directions of M enterprises, this paper shows that the proportion of different industrial directions in the LCE is different, and the proportion of different industries in different low-carbon economic indicators is also different. Therefore, in order to reasonably promote the rational development of the sports industry of enterprises and meet the needs of the current social low-carbon economic development, enterprises should reasonably dominate the development of various industries to provide impetus for the overall development of enterprises. Table 1 shows the regression analysis results of the development of sports industry under the low-carbon economic structure.

**Table 1:** Analysis of regression results.

Low carbon economy indicators	Overall sports industry	Sports service industry	Sports manufacturing industry	Construction of sports venues and facilities
a	0.441^*^	0.745	0.542^*^	0.648^*^
b	0.184	0.115	0.824	0.959
c	0.541^*^	0.087^*^	0.797	0.935^*^
d	0.111	0.904	0.211^*^	0.251
e	0.632	0.249	0.718	0.128
f	0.837^*^	0.193^*^	0.915^*^	0.819^*^
g	0.651^*^	0.351^*^	0.687^*^	0.57^*^
h	0.699^*^	0.793^*^	0.874^*^	0.058^*^
i	0.908	0.901	0.854^*^	0.716
j	0.448^*^	0.761	0.032	0.677
k	0.968	0.816^*^	0.266^*^	0.957
l	0.303^*^	0.369	0.256	0.224

^*^Indicates significant results.

In Table 1, many indicators show that the current results are significant, which shows that low-carbon economic indicators have a significant impact on the development of sports industry, so the development of low-carbon economic structure is the course of sports industry reform and development.

### 4.3. Discussion

In order to promote the development of sports industry under the low-carbon economic structure, this paper studies the development of sports industry based on the low-carbon economic structure, and evaluates the influence of the current low-carbon economic structure on the development of sports industry by designing low-carbon economic indicators. The results show that the current low-carbon economic structure has a significant impact on the development of sports industry, because the whole society is developing towards low-carbon economic structure, and sports industry is no exception. In addition, under the background of low-carbon economic development, improving the public health strategy is also necessary. Only by striving to improve public health can people guarantee residents’ quality of life for a long time and promote the development of society. Therefore, to promote the development of social public health system, this paper puts forward the following suggestions for improving public health:(1) Formulate public policies to promote the perfection of laws and regulations. The development of LCE must be guaranteed by laws and regulations first, so China should formulate corresponding perfect laws and regulations according to China’s specific national conditions to ensure the smooth development of LCE from the legislative aspect. By analyzing the specific situation of various industries in China, people should restrict enterprises with serious pollution and high energy consumption, and formulate perfect laws and regulations to escort the development of LCE. People should also constantly improve and revise the legal system according to the changing social situation to ensure the development of LCE in a better direction. (2) Formulate public policies to promote the development of low-carbon technologies. First, the state should formulate corresponding public economic policies, vigorously support the development of low-carbon technologies, and allocate certain funds for the special development of low-carbon technologies to provide a better guarantee for the development of low-carbon technologies. People can learn from developed countries’ low-carbon technologies and development models, learn from their advantages, and make bold innovations to create low-carbon technologies and promotion models suitable for China ‘s specific national conditions, and enhance the adaptability of low-carbon technologies in China. People can also support and encourage the development of low-carbon energy and low-carbon enterprises by formulating some preferential policies, and encourage enterprises to develop low-carbon technologies. (3) Reform and improve the legal guarantee system of public health. People should conscientiously implement the laws and regulations on emergency management of public health emergencies, improve the management systems of unpaid blood donation and social medical first aid, improve the epidemic prevention and control mechanism with clear rights and responsibilities, standardized procedures and strong implementation, and clarify matters such as incident reporting, epidemic disposal, material reserves, social expropriation, social organizations and public responsibilities.

## 5. Conclusion

With the continuous development and progress of society, the direction of social industrial production technology is constantly optimized, so to promote society’s sustainable development, the green economy model has become the main direction of social industrial production development. Based on this, in order to promote the sustainable development of sports industry under the background of green economy and improve the comprehensive level of public health strategy, firstly, this paper introduces the current development of green economy. Then the basic composition and development status of sports industry are discussed. Finally, based on the background of green economy, the current development status of sports industry and the evolution status of public health level are analyzed, and corresponding development suggestions are put forward. The results show that the current social sports industry has broad prospects for development. In 2020, the sports industry will increase by 11.6% year-on-year, accounting for 1.14% of GDP. The proportion of added value of sports industry in GDP is increasing year by year, which shows that sports industry plays a significant contribution to economic growth. As for the development of sports industry in a LCE, different branches of the industry are facing different problems. Among them, the output rate of fixed assets, technical level and labor productivity are better, while other indicators are weaker. Therefore, the development of sports industry needs to carry out targeted reforms in each branch to promote the comprehensive development of sports industry. As for the development of sports industry in a LCE, different industry branches face different problems. Among them, the output rate of fixed assets, technical level and labor productivity are better, while other indicators are weaker. Therefore, the development of sports industry needs to carry out targeted reforms in each branch to promote the comprehensive development of sports industry.

Although this paper provides suggestions for developing the sports industry and improving public health strategies, the current research only studies M enterprises, so the representativeness of the research results is not high. Based on this, the research scope will be expanded and the number of research samples will be increased in the future research, thus improving the value of this paper.

## Data availability statement

The raw data supporting the conclusions of this article will be made available by the authors, without undue reservation.

## Ethics statement

The studies involving human participants were reviewed and approved by Sichuan University Ethics Committee. The patients/participants provided their written informed consent to participate in this study. Written informed consent was obtained from the individual(s) for the publication of any potentially identifiable images or data included in this article.

## Author contributions

All authors listed have made a substantial, direct and intellectual contribution to the work, and approved it for publication.

## Conflict of interest

The authors declare that the research was conducted in the absence of any commercial or financial relationships that could be construed as a potential conflict of interest.

## Publisher’s note

All claims expressed in this article are solely those of the authors and do not necessarily represent those of their affiliated organizations, or those of the publisher, the editors and the reviewers. Any product that may be evaluated in this article, or claim that may be made by its manufacturer, is not guaranteed or endorsed by the publisher.
